# Vulvar Necrosis Following Uterine Artery Embolisation for Uterine Fibroid Treatment: A Case Report and Literature Review

**DOI:** 10.7759/cureus.101190

**Published:** 2026-01-09

**Authors:** Hesham Mahmoud, Doaa Mohammed, Iheoma Amaechi

**Affiliations:** 1 Obstetrics and Gynaecology, Medway Maritime Hospital, Gillingham, GBR; 2 Obstetrics and Gynaecology, St George's University Hospitals NHS Foundation Trust, London, GBR; 3 Interventional Radiology, Medway NHS Foundation Trust, Medway, GBR

**Keywords:** benign gynaecology, heavy menstrual loss, interventional radiology guided embolization, uterine fibroid, uterine fibroid embolization

## Abstract

Uterine artery embolisation (UAE) is a recognised minimally invasive treatment for symptomatic uterine fibroids, with a generally favourable safety profile. Although post-embolisation complications are well documented, vulvar necrosis is exceptionally rare.

We report the case of a 50-year-old woman of Black African heritage who developed severe vulvar symptoms eight days after UAE was undertaken for symptomatic multiple fibroids. She presented with intense vulvar pain, swelling, erythema, ulceration, and right-sided gluteal skin changes, alongside numbness in the lateral right leg.

Examination revealed ulceration and sloughing of the right vulva, with progression to gluteal necrosis over seven days. CT and MRI imaging demonstrated inflammatory changes and infarction of the subcutaneous fat in the right buttock, consistent with non-target embolisation. Multidisciplinary assessment concluded that the findings were due to extravasation of embolic material into the gluteal region.

The patient received broad-spectrum intravenous antibiotics, neuropathic pain management, and multidisciplinary care involving gynaecology, surgery, interventional radiology, pain management, neurology, and tissue viability teams. She underwent surgical debridement of the necrotic vulvar tissue, achieving full healing. The gluteal necrosis was managed conservatively with spontaneous resolution. At three-month follow-up, she was asymptomatic from a gynaecological perspective; at two years, she was diagnosed with right leg pain syndrome, possibly piriformis-related.

Vulvar necrosis following UAE is an exceptionally rare but clinically significant complication. This case highlights the need for early recognition of post-procedural vulvar or gluteal changes and rapid multidisciplinary intervention to optimise outcomes. Increased reporting of such cases may help clarify risk factors, refine preventive strategies, and guide management.

## Introduction

Uterine artery embolisation (UAE) has emerged as an effective minimally invasive alternative to surgical interventions for the treatment of symptomatic uterine fibroids [1]. Since its introduction in the 1990s, the UAE has gained popularity due to its advantages, including shorter hospital stays, quicker recovery times, and preservation of the uterus [2]. The procedure involves occlusion of the uterine arteries that supply blood to the fibroids, leading to ischaemic infarction and subsequent shrinkage of the fibroids [3].

Uterine artery embolisation (UAE) is used for a range of benign gynaecological and obstetric conditions. Its primary indication is symptomatic uterine fibroids, but it is also employed for adenomyosis, uterine arteriovenous malformations, and selected cases of acute or postpartum haemorrhage [4-6]. UAE can additionally support conservative management in abnormal placentation and other sources of pelvic bleeding when fertility preservation or minimally invasive treatment is preferred [7].

While the UAE is generally considered safe with high success rates, it is associated with various complications. These range from minor issues such as post-embolisation syndrome (characterised by pain, fever, nausea, vomiting, and malaise) to more serious complications, including infection, non-target embolisation, and, rarely, tissue necrosis [8,9]. The reported rates of complications vary, with approximately 5-7% for major complications within the first year after UAE [10].

Tissue necrosis following UAE is one of the rarest complications, with uterine necrosis being reported in fewer than 30 cases in medical literature since the advent of UAE [11]. Vulvar necrosis specifically is even more uncommon, with extremely limited documentation. Pathophysiology is thought to involve excessive embolisation, use of very small particles (<500 microns), and lack of collateral blood supply to the affected tissues [12]. The clinical significance of vulvar necrosis lies in its potential for serious morbidity, including sepsis, and the diagnostic and management challenges it presents to clinicians. Atypical vulvar findings can initially be mistaken for common benign conditions such as infection, Bartholin abscess, inflammation, or minor trauma, which may delay recognition of uncommon underlying causes [13]. This overlap can lead to delayed recognition of uncommon underlying causes, particularly when symptoms do not follow the expected pattern.

Our case report documents a rare occurrence of vulvar necrosis, which presented 10 days after UAE for fibroid treatment, mainly with ulceration in the right vulvar and outer vaginal region and discolouration of the right gluteal region. Imaging played an important role, including ultrasound, which often is the initial modality, followed by CT or MRI for confirmation. A multidisciplinary approach was followed throughout the management. This case adds to the limited body of knowledge on this complication and highlights important diagnostic and clinical considerations for practitioners performing UAE.

## Case presentation

A 50-year-old female patient of Black African heritage with multiple fibroids was initially assessed in the gynaecology outpatient clinic with heavy menstrual bleeding and pressure symptoms for the past 15 years. She reported regular periods but with long and heavy bleeding, lasting up to two weeks. Examination revealed a 20-week-sized uterus with limited mobility. Ultrasound and MRI imaging were requested.

**Figure 1:**
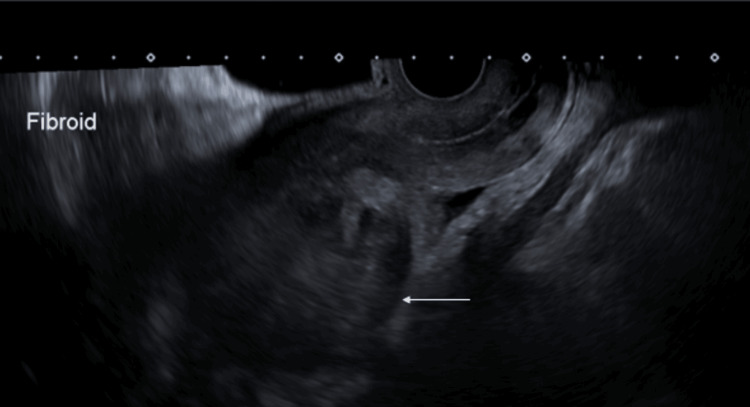
Pre-embolisation transvaginal ultrasound image in the sagittal plane depicting a fundal fibroid (arrow)

**Figure 2:**
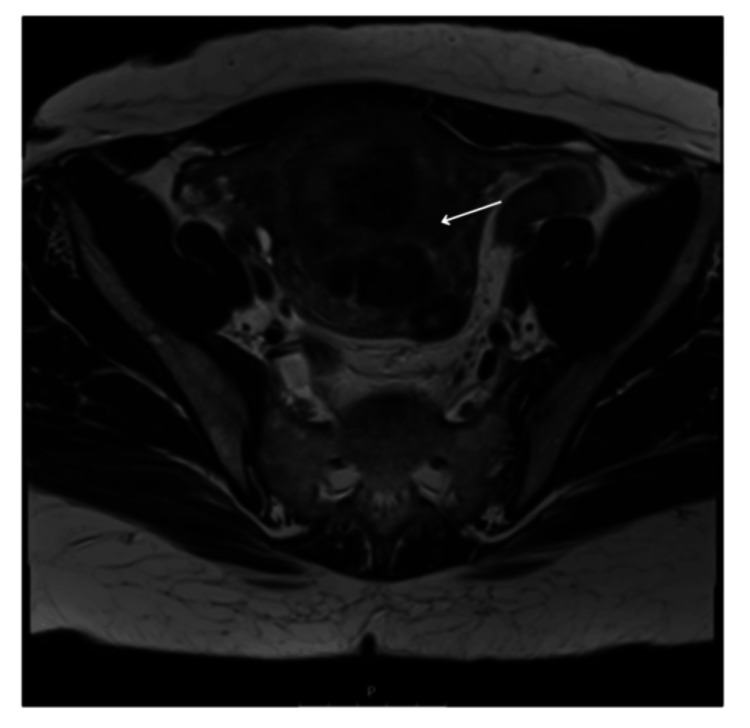
Axial large field of view T2 weighted MRI image of the pelvis showing uterine fibroids (arrow), pre-embolisation

**Figure 3:**
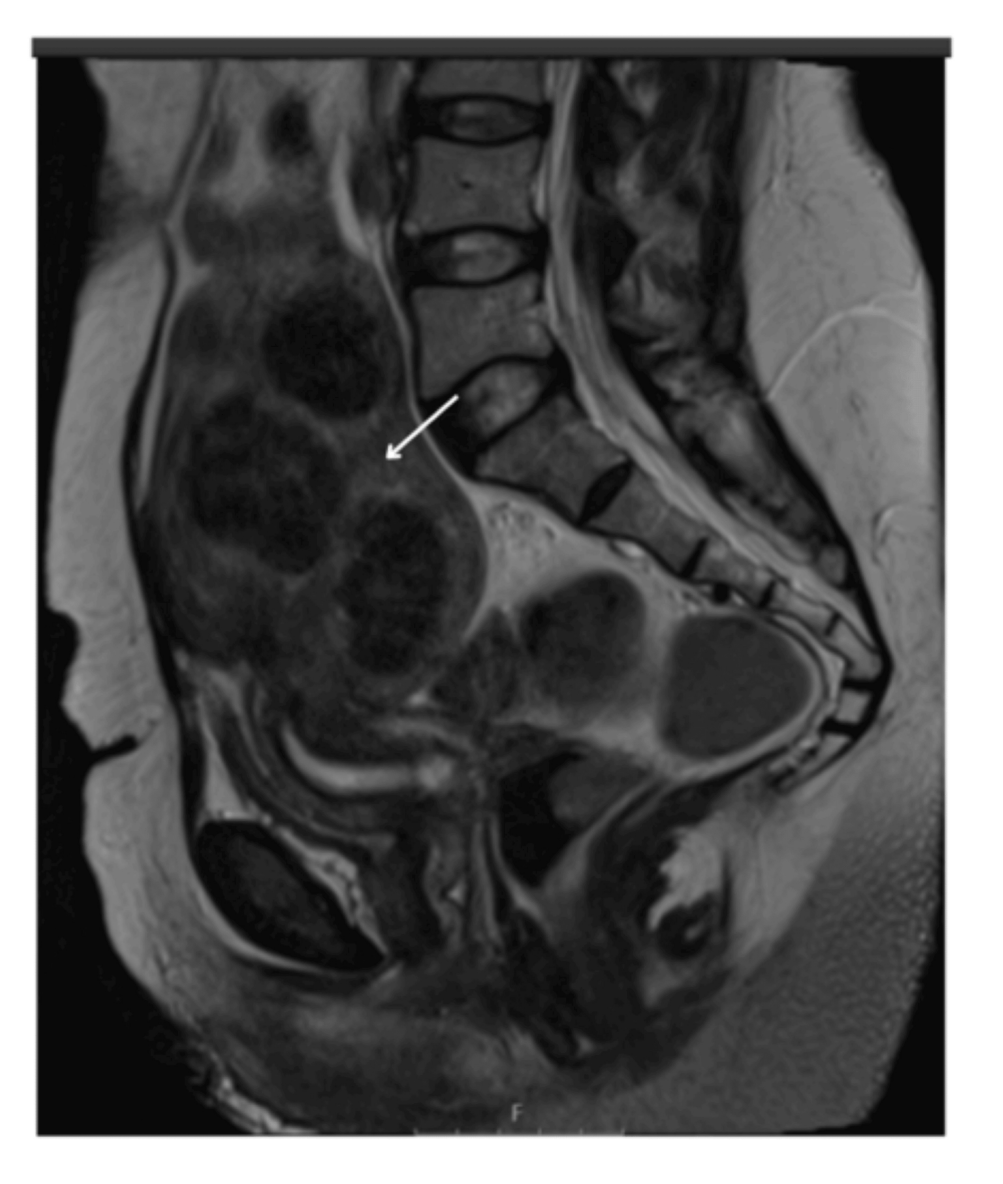
Sagittal T2 weighted large field of view MRI image of the pelvis showing fibroid uterus (arrow), pre-embolisation

The patient's imaging was reviewed, including MRI. An ultrasound showed an 18 cm fibroid encroaching on the uterine cavity (Figure 1). An MRI has been performed and confirmed multiple uterine fibroids (Figures 2, 3). Different management options were discussed with the patient, including UAE. A decision was made for the UAE and referred to Interventional Radiology (IR), and the decision of the UAE was confirmed.

**Figure 4:**
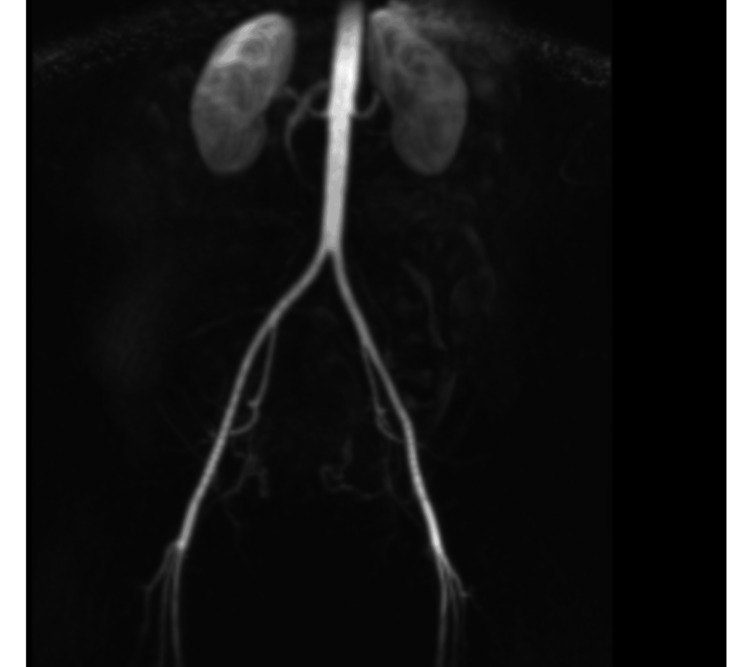
MRI image of dynamic contrast enhanced pelvic MRI angiogram depicting opacification of bilateral uterine arteries with no obvious variant uterine arterial supply

The UAE procedure was performed under local anaesthesia. A right common femoral artery retrograde puncture was performed under ultrasound guidance, and a 5 French sheath was placed. The aortic bifurcation was crossed with a 5 French contra catheter, and a guidewire was manipulated into the left internal iliac artery. The ipsilateral right internal iliac artery was selectively catheterised with the contra catheter, and the right uterine artery was cannulated with the Progreat micro catheter. The right uterine artery was embolized with a combination of 700- and 900-micrometer embosphere particles (Varian - Siemens Healthineers Company) until stasis was achieved. The ipsilateral right internal iliac artery was selectively catheterised with the contra catheter, and the right uterine artery was cannulated with the Progreat micro catheter. The right uterine artery was embolised with a combination of 700 and 900 micrometer embolization particles until stasis was achieved. Haemostasis at the right groin puncture site was achieved with deployment of a 6-French Angio-Seal device. No immediate complications were noted. Post-procedure contrast-enhanced pelvic MR angiogram showed no abnormal findings. Figure 4 shows an MRI image of a dynamic contrast-enhanced pelvic MRI angiogram depicting opacification of bilateral uterine arteries with no obvious variant uterine arterial supply.

**Figure 5:**
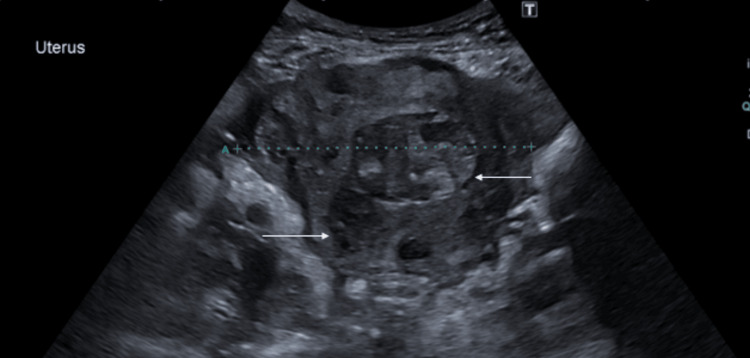
Post-embolisation trans-abdominal US pelvis in transverse plane depicting uterine fibroids (arrows)

Figure 5 shows post-embolisation ultrasound image in the sagittal plane depicting a uterine fibroid. 

**Figure 6:**
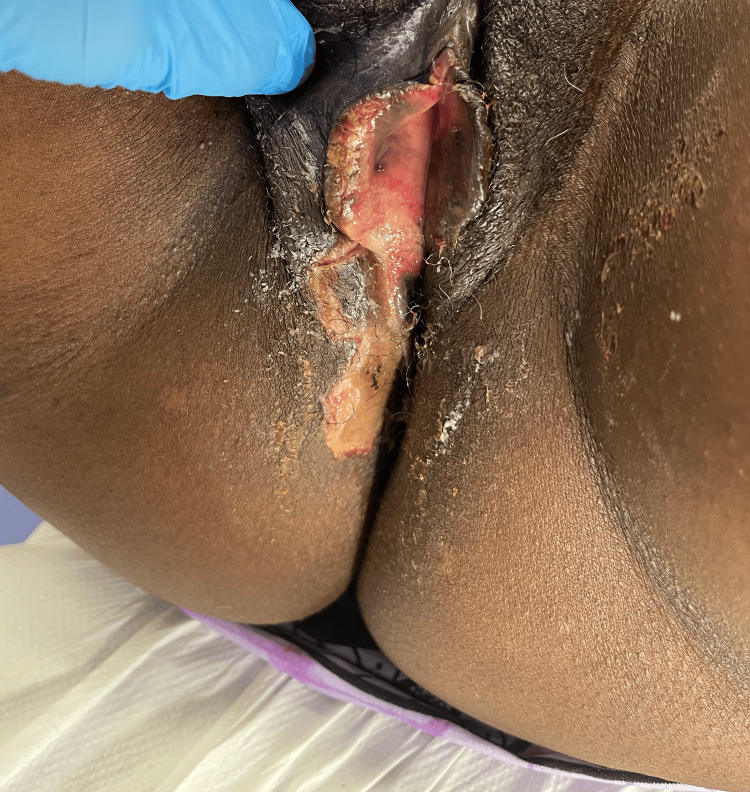
Right vulvar necrotic skin patch on admission

**Figure 7:**
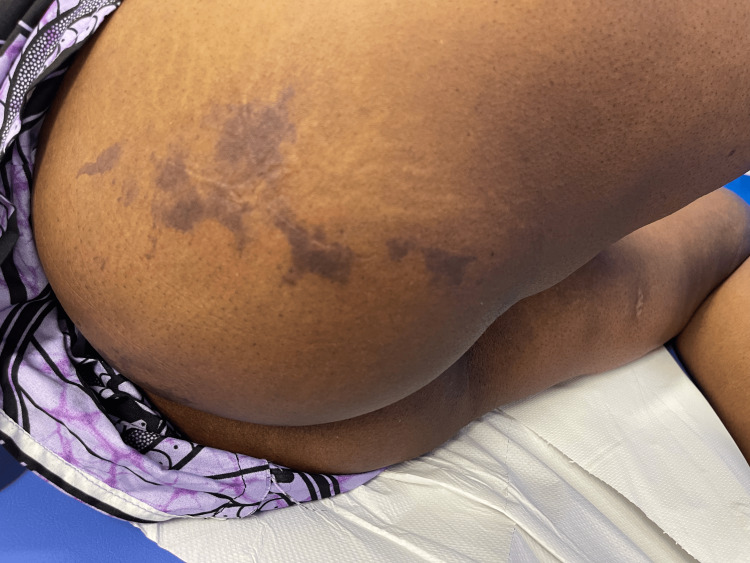
Right gluteal necrotic skin patch on day of admission

Eight days post procedure: The patient was admitted to the gynaecology ward with vulvar pain, soreness, swelling, redness, and ulceration involving the right vulva and outer vaginal region, accompanied by numbness along the lateral aspect of the right leg. On examination, there was an irregular ulcerative lesion involving the right vulvar region, measuring approximately 5 × 3 cm (Figure 6). The central area showed exposed red, moist tissue consistent with breakdown, surrounded inferiorly by a pale yellow-tan slough with patches of yellow-coloured necrotic surface. The margins were sharply demarcated with dry, darkened tissue at the edges. Surrounding vulvar skin appeared intact but mildly inflamed, without blistering, discharge, or crepitus. The patient reported significant tenderness on palpation. Additionally, well-demarcated interrupted areas of dusky purple-black discolouration involving the right gluteal region, the largest of which measures approximately 10 × 8 cm, were noted. The surface appeared dry and non-blanching, features consistent with early ischaemic change (Figure 7), which progressed to necrosis after seven days. The vulvar changes appeared non-specific and resembled common benign conditions, making early diagnosis challenging.

**Figure 8:**
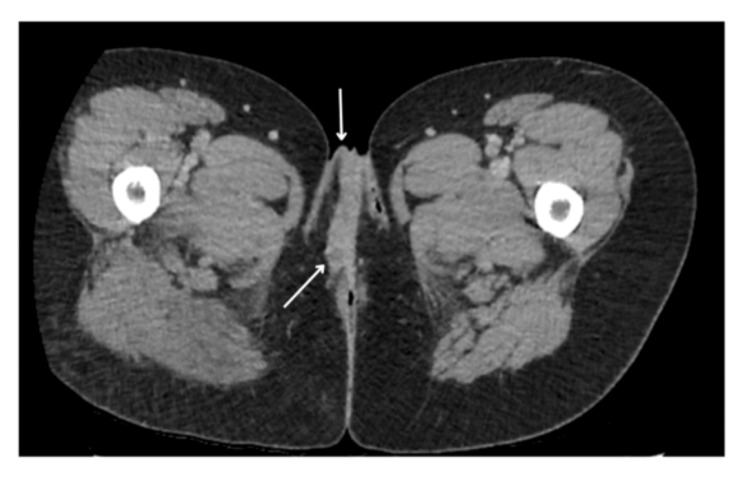
Axial post-contrast venous phase CT scan of the pelvis following presentation with complications post-embolisation. Thickening of the right labia consistent with oedema and inflammation (arrows)

**Figure 9:**
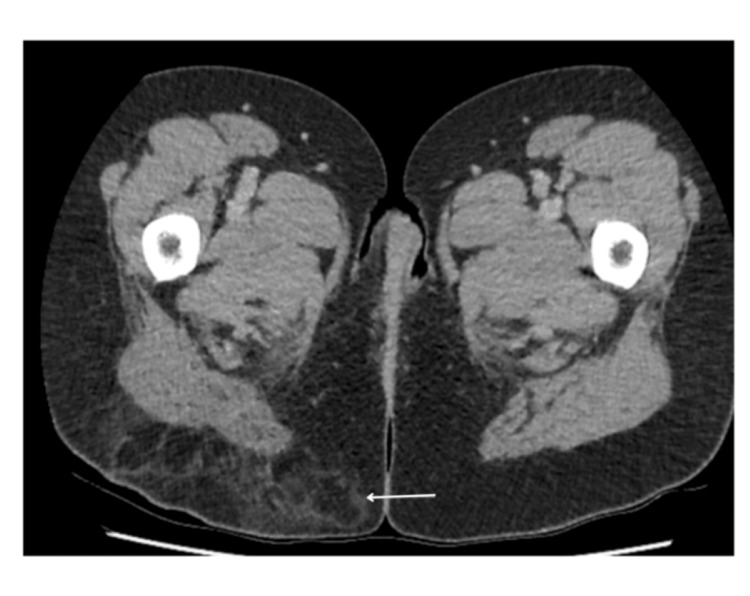
Axial post-contrast venous phase pelvic CT scan. Progression of appearances within the subcutaneous fat overlying the right buttock showing marked inflammatory fat stranding (arrow)

**Figure 10:**
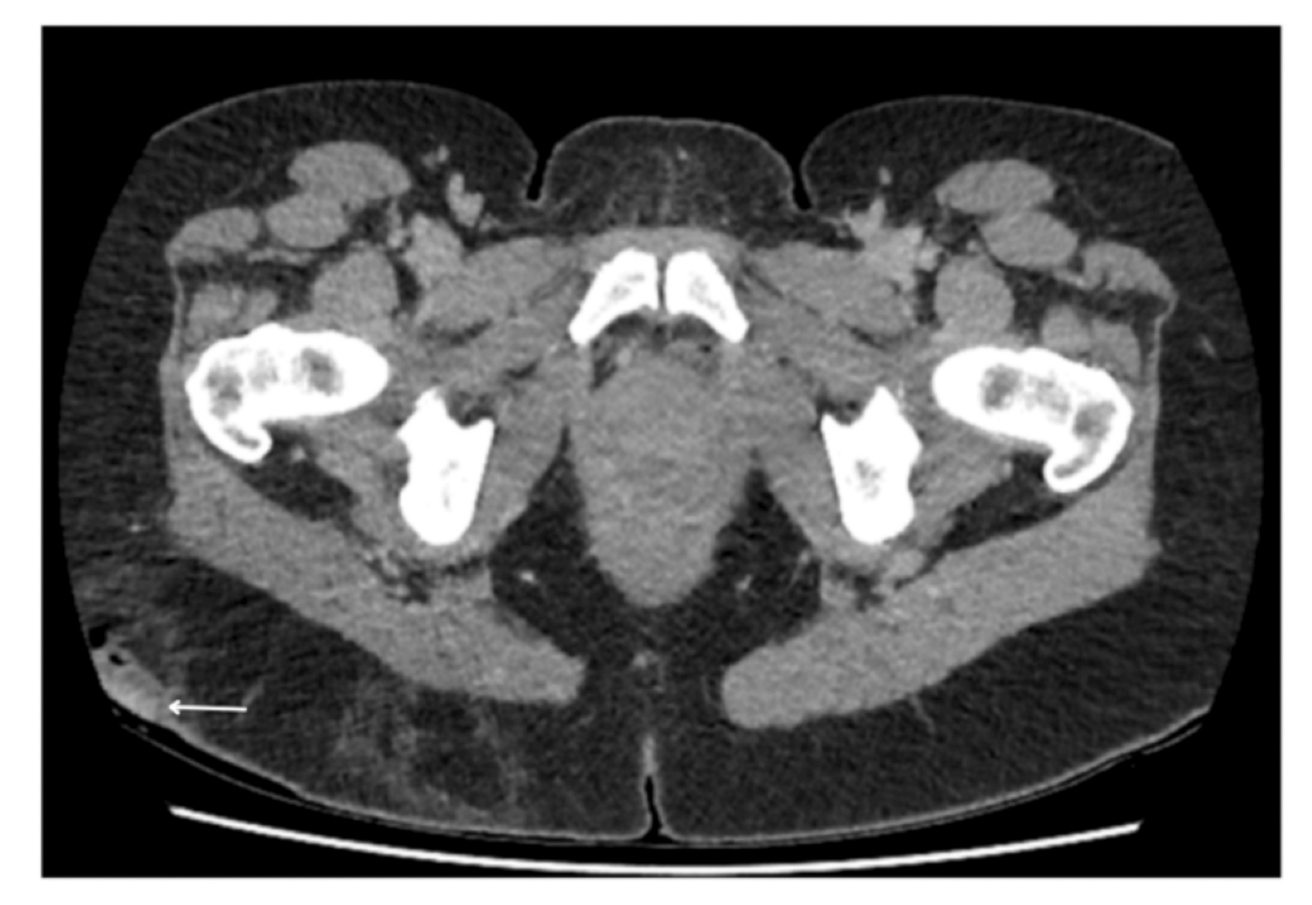
Axial post-contrast venous phase CT scan image of the pelvis. Small area of subcutaneous density containing small gas pocket in keeping with fat necrosis (arrow)

The patient underwent CT and MRI scans for further investigations of the lesions. Figure 8 shows a contrast venous phase CT scan of the pelvis showing thickening of the right labia. Figures 9, 10 are axial post-contrast venous phase pelvic CT scans showing progression of appearances within the subcutaneous fat overlying the right buttock with marked inflammatory fat stranding and subcutaneous density containing small gas pockets in keeping with fat necrosis, respectively.

**Figure 11:**
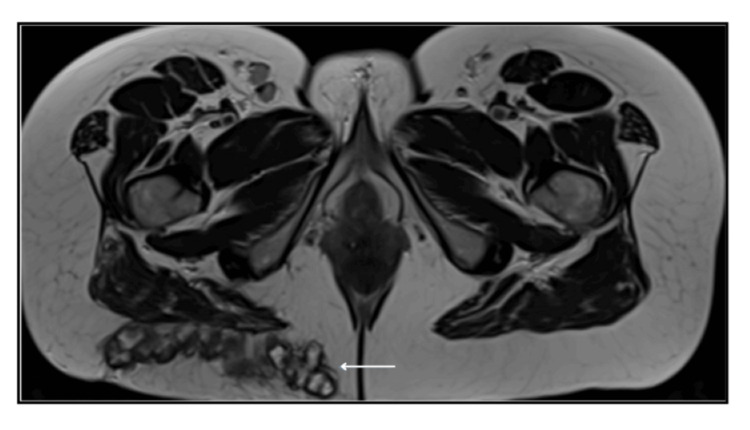
Axial T2W large field of view MRI image of the pelvis post embolisation. Infarction of subcutaneous fat within the right buttock with thick-walled encapsulated fat lobules (arrow)

**Figure 12:**
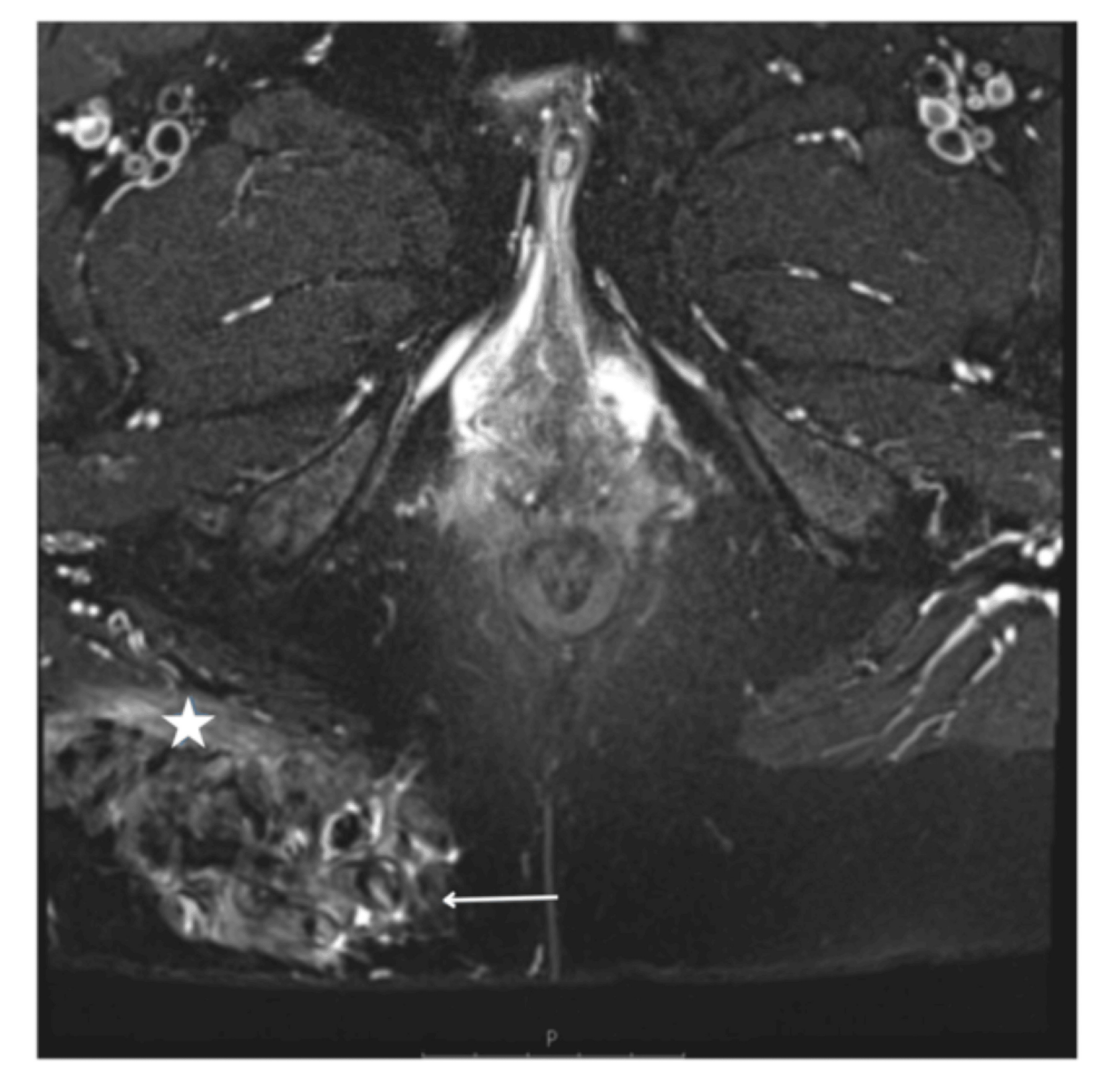
Axial T2 fat sat MRI image of the pelvis. Inflammatory stranding and thickening surrounding fat lobules with the right buttock (arrow). Inflammatory, oedematous signal also involving the adjacent right gluteal muscle fibres (asterisk)

MRI images of the pelvis were also done for further investigation of the lesions, showing fat infarction and associated inflammatory stranding surrounding the adjacent fat lobules, demonstrating subcutaneous fat necrosis in the buttock region (Figures 11, 12).

The patient was reviewed and discussed with the surgical team for multidisciplinary management, and intravenous antibiotics were initiated on admission. The multidisciplinary team was later expanded to include the Pain Management Team, Interventional Radiology, the Tissue Viability Team, and Neurology. Pregabalin was commenced for neuropathic pain control. The patient was referred to the neurology team for paraesthesia in the right foot. Spine MRI findings were unremarkable, and nerve conduction studies were requested. The condition was subsequently diagnosed as extravasation of embolic material into the right gluteal region, resulting in skin sloughing.

The patient underwent examination under anaesthesia and had surgical debridement for the necrotic vulvar area with uneventful full healing. Conservative management was undertaken for the right gluteal region to allow spontaneous healing, as the necrosis was superficial.

**Figure 13:**
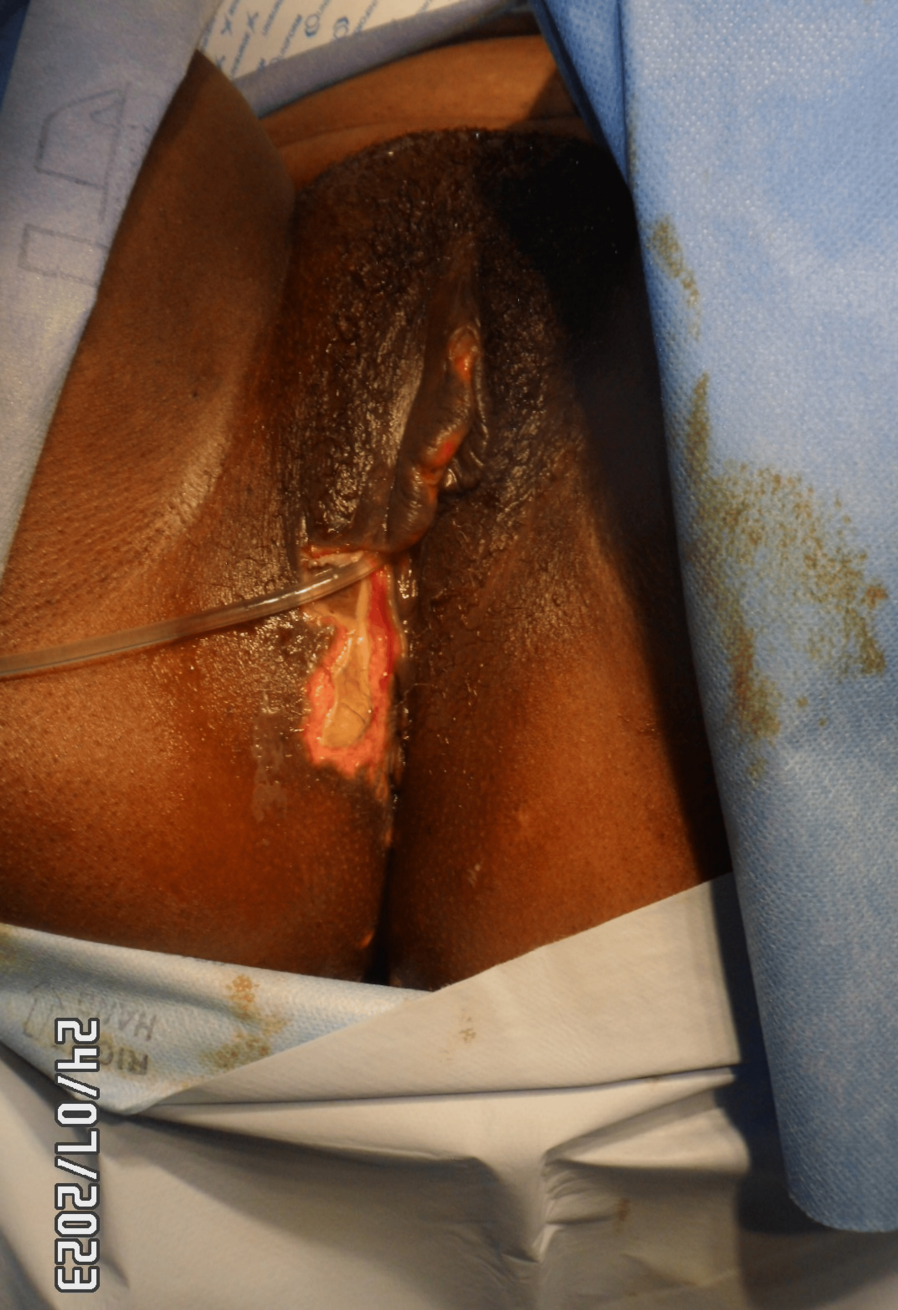
Examination under anaesthesia for the right vulvar necrotic skin patch

**Figure 14:**
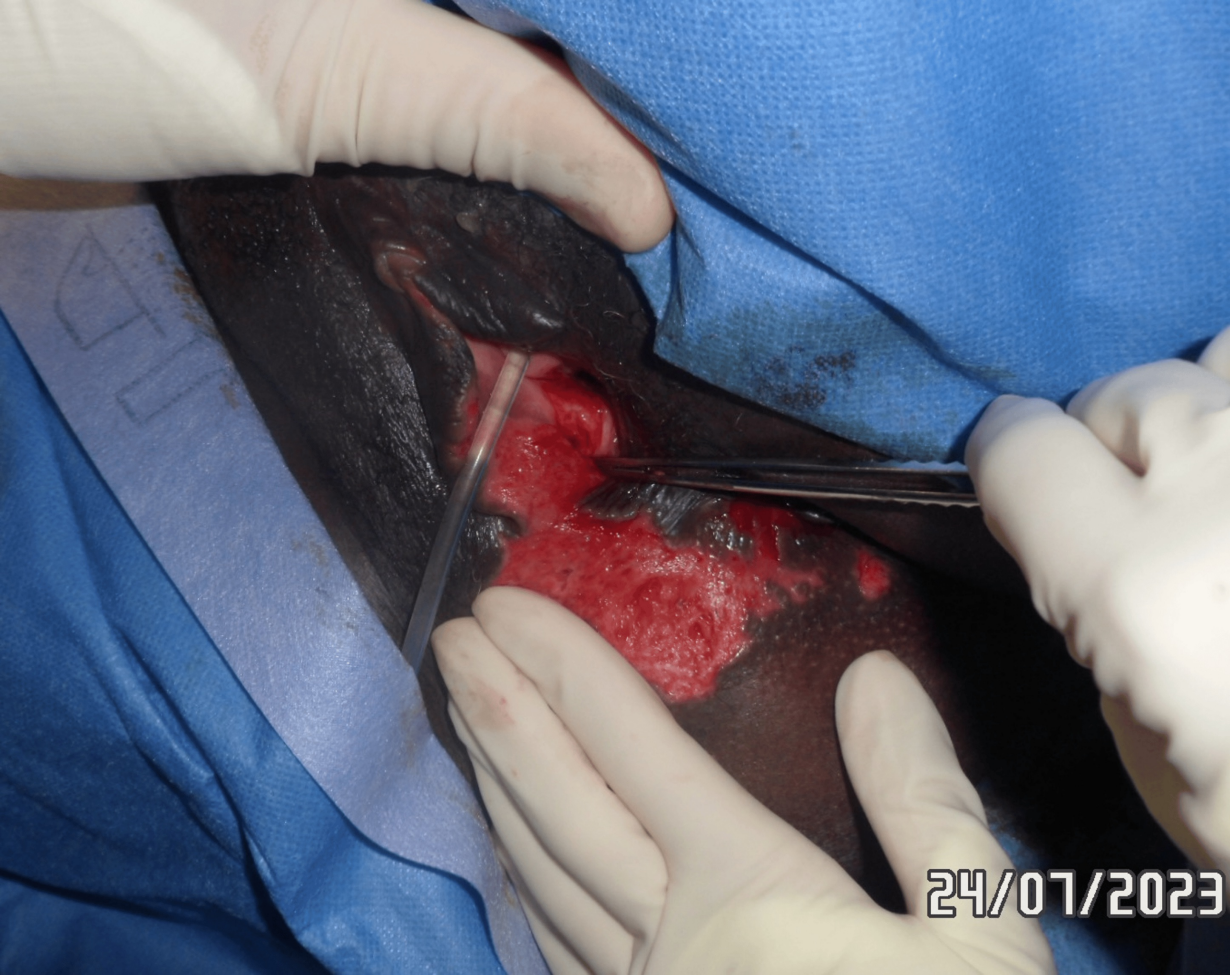
Right vulva after surgical debridement of the necrotic skin patch

**Figure 15:**
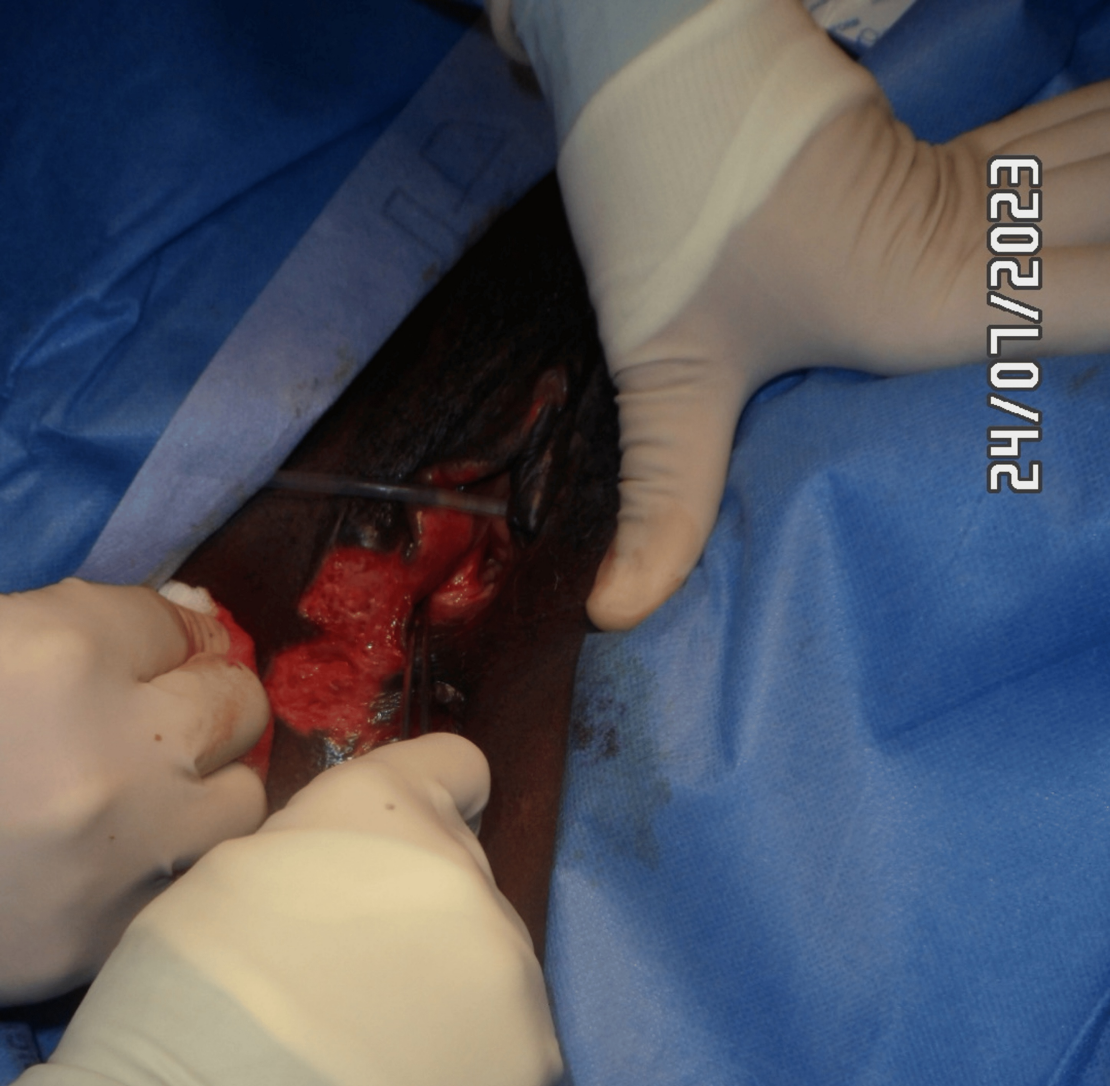
Right vulva after debridement of the necrotic skin patch

**Figure 16:**
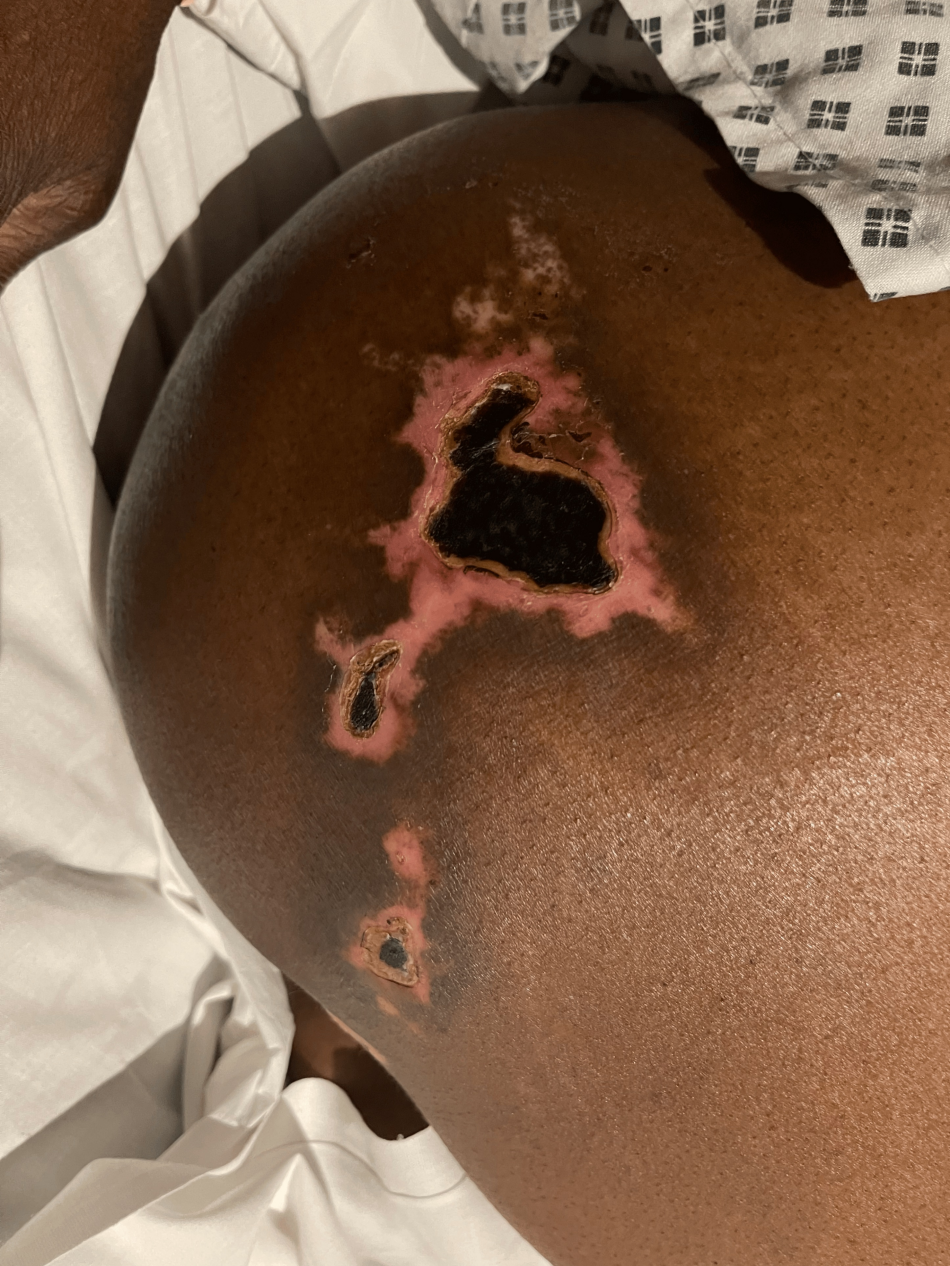
Right gluteal necrotic skin patch 10 days after admission

Figure 13 shows a right vulvar necrotic lesion intra-operatively during examination under anaesthesia. Figures 14, 15 show the right vulva after surgical debridement of the necrotic skin lesion. Figure 16 shows a right gluteal necrotic skin patch 10 days after admission.

The patient was seen three months post procedure in the gynaecology outpatient clinic for follow-up and discharged, as there were no gynaecological symptoms. The patient remained under follow-up for UAE complications. Two years post-procedure, she was diagnosed with right leg syndrome, possibly piriformis syndrome.

## Discussion

Vulvar necrosis following uterine artery embolisation represents an extremely rare but serious complication that warrants careful consideration. This case adds to the limited literature on this specific complication and provides insights into its presentation, diagnosis, and management.

Pathophysiology of vulvar necrosis after UAE: The pathophysiology of tissue necrosis following UAE is multifactorial. Several theories have been proposed to explain its occurrence. First, the use of very small embolic particles (<500 microns) may lead to more distal occlusion of vessels, potentially affecting collateral circulation and causing more extensive ischaemia than intended [14,15]. In our case, embozene particles of 700 and 900 micrometres were used.

Second, the lack of adequate collateral blood supply to embolised regions plays a crucial role. The uterus typically has rich collateral circulation through the ovarian, vaginal, and other pelvic vessels. However, anatomical variations or previous surgeries may compromise these collateral pathways, increasing the risk of necrosis [16]. Our patient did not have specific factors identified that might have compromised collateral circulation.

Third, technical aspects of the embolisation procedure itself may contribute to necrosis. Unselective embolisation and embolisation until complete stasis is achieved (rather than near-stasis) have been identified as technical risk factors [17]. In the present case, the left uterine artery embolisation with embozene particles was performed according to the manufacturer's recommendation until stasis was achieved.

The specific involvement of vulvar structures in necrosis is particularly rare and may relate to their unique blood supply and vulnerability to ischaemic damage. Vulvar structures in the female reproductive tract receive blood supply primarily from branches of the uterine artery with limited collateral circulation [18].

The clinical presentation of tissue necrosis after UAE typically includes persistent or worsening abdominal pain, fever, malodorous vaginal discharge, and signs of infection or sepsis [19]. In our case, the patient presented with vulvar pain, soreness, swelling, redness, and numbness on the lateral aspect of the left leg, along with ulceration and sloughing of the vulva and right gluteal region, just eight days after the procedure. This is consistent with the timeframe reported in literature, where the interval between UAE and diagnosis of uterine necrosis ranges from 4 to 69 days [20].

Diagnosis of vulvar necrosis presents challenges due to its rarity and potentially nonspecific initial symptoms. Imaging plays a crucial role, with ultrasound often serving as the initial modality, followed by CT or MRI for confirmation. MRI findings may include a lack of myometrial enhancement, myometrial gas, and peripheral contrast uptake, which indicate myometrial necrosis [21]. In our patient, MRI of the right foot showed no abnormalities, but diagnostic imaging of the affected vulvar and gluteal regions revealed the extent of the necrosis.

Laboratory markers of inflammation and infection, including elevated white blood cell count, C-reactive protein, and erythrocyte sedimentation rate, are typically present but nonspecific [22].

Management of vulvar necrosis following UAE requires a multidisciplinary approach. Treatment options range from conservative management with broad-spectrum antibiotics to surgical intervention, depending on the extent of necrosis and the patient's clinical condition [23].

Conservative management may be attempted in haemodynamically stable patients with limited necrosis and includes intravenous antibiotics, pain management, and close monitoring [24]. However, surgical intervention is often necessary in cases of extensive necrosis or sepsis. Surgical options include debridement of necrotic tissue, hysteroscopic resection, or hysterectomy in severe cases [25].

In our case, the patient underwent surgical debridement for the vulvar region with full healing eventually, while the gluteal region was managed conservatively. This approach was chosen based on the extent of the necrosis and the need for tissue salvage.

Several strategies have been proposed to minimise the risk of tissue necrosis following UAE. Careful patient selection, with consideration of anatomical factors and previous surgeries that might affect collateral circulation [14]. Technical considerations during the procedure, including the use of larger embolic particles (>500 μm), avoiding embolisation to complete stasis, and ensuring selective catheterisation [26]. Furthermore, prophylactic antibiotics, although their role in preventing necrosis specifically (rather than infection generally) remains unclear [27]. Close post-procedural monitoring with prompt investigation of persistent or worsening symptoms [28].

A review of the literature reveals fewer than 30 reported cases of uterine necrosis following UAE, with vulvar necrosis being even rarer [29]. Mutiso et al. (2018) documented a case of uterine necrosis one month after UAE for symptomatic fibroids, with the patient presenting with abdominal pain and vaginal discharge [11]. Similarly, Han et al. (2022) reviewed 21 cases of uterine necrosis following UAE and found that the most common clinical symptoms were fever, abdominal pain, and vaginal discharge [20].

In addition to these cases, Yeagley et al. (2002) reported a case of labial necrosis following UAE for leiomyomata, in which a 38-year-old woman developed a 3 cm necrotic lesion of the right labium minora that resolved spontaneously over four weeks with conservative therapy [30]. The authors attributed the necrosis to retrograde reflux of embolic material into the internal pudendal artery, emphasising the potential for collateral flow between the uterine and pudendal arterial systems and the need for meticulous embolisation technique. This case further supports the concept that vulvar tissues may be vulnerable to non-target embolisation despite UAE generally being considered safe.

Our case is distinctive in several aspects: the rapid development of vulvar necrosis within 10 days is particularly noteworthy, as most reported cases have a longer interval between UAE and the diagnosis of necrosis. Additionally, the involvement of the vulva and right gluteal region, coupled with neurological symptoms, presents a complex clinical picture.

This case highlights several important considerations for clinical practice. Clinicians should maintain a high index of suspicion for tissue necrosis in patients presenting with persistent pain, fever, or malodorous discharge following UAE, even within a relatively short timeframe after the procedure. Prompt diagnostic imaging is crucial when necrosis is suspected, with MRI being particularly valuable for assessing the extent of tissue damage. A multidisciplinary approach involving interventional radiologists, gynaecologists, and microbiologists may optimise patient outcomes when managing this rare complication. Furthermore, thorough pre-procedure counselling should include discussion of this rare but serious potential complication, particularly in patients with factors that might increase their risk.

## Conclusions

Vulvar necrosis following uterine artery embolisation for fibroid treatment is an extremely rare complication with significant clinical implications. This case report documents its occurrence in a female patient with Black African heritage within 10 days of the procedure, highlighting the importance of vigilance in the post-procedural period. While the UAE remains a valuable treatment option for symptomatic fibroids with a generally favourable safety profile, awareness of potential complications such as vulvar necrosis is essential for timely diagnosis and management. Early evaluation of any atypical vulvar symptoms after pelvic procedures may therefore play a crucial role in preventing delays in recognition. Further research and documentation of such cases will continue to enhance our understanding of risk factors, preventive strategies, and optimal management approaches.
